# Supplementary material for: High level non-carbapenemase carbapenem resistance by overlaying mutations of mexR, oprD, and ftsI in Pseudomonas aeruginosa
Source: Microbiol Spectr. 2024 Nov 18;13(1):e01398-24. doi: 10.1128/spectrum.01398-24 (PMC11705820; doi:10.1128/spectrum.01398-24)
Supplement: Supplemental material — Tables S1 to S3; Fig. S1 and S2. [file spectrum.01398-24-s0002.docx]

**Supplementary Information**

**Supplementary Tables**

**Table S1. Strains and plasmids used in this study.**

| **Strain or plasmid** | **Description** | **Reference** |
| --- | --- | --- |
| Trelief® 5α | Chemically competent cell of *E. coli* DH 5α mutant with high transformation efficiency | TSINGKE |
| BL21(DE3) | Chemically competent cell of *E. coli* BL21(DE3) | TSINGKE |
| Pae d1 Green | Green pigment producing, carbapenem-susceptible, isolated on November 25, 2020 | This study |
| Pae d1 Brown | Brown pigment producing, carbapenem-susceptible, isolated on November 25, 2020 | This study |
| Pae d14 Green | Green pigment producing, carbapenem-resistant, isolated on December 8, 2020 | This study |
| Pae d14 Brown | Brown pigment producing, carbapenem-resistant, isolated on December 8, 2020 | This study |
| Pae d17 Green | Green pigment producing, carbapenem-resistant, isolated on December 11, 2020 | This study |
| Pae d17 Brown | Brown pigment producing, carbapenem-resistant, isolated on December 11, 2020 | This study |
| *P. aeruginosa* ATCC 27853 | Quality control strain for MIC determination | Laboratory storage |
| *P. aeruginosa* PAO1 | Model strain | Laboratory storage |
| PAO1 *∆mexR* | *P. aeruginosa* PAO1 which *mexR* gene deleted | This study |
| PAO1 *∆mexR* pUCP18-*mexR* | PAO1 ∆*mexR* complemented with pUCP18 plasmid carrying wild-type *mexR* | This study |
| PAO1 *∆mexR* pUCP18-*mexRA164C* | PAO1 ∆*mexR* complemented with pUCP18 plasmid carrying mutated *mexR* | This study |
| PAO1 *∆mexR* pUCP18-empty | PAO1 ∆*mexR* complemented with empty pUCP18 plasmid | This study |
| Pae d1 Green oprD415* | Pae d1 Green with OprD415* mutation | This study |
| Pae d1 Green ∆*mexR* | Pae d1 Green which *mexR* gene deleted | This study |
| Pae d1 Green oprD415*∆*mexR* | Pae d1 Green with both OprD415*mutation and *mexR* gene deleted | This study |
| Pae d1 Green ∆oprD::WT oprD | Pae d1 Green ∆oprD415 in-situ complemented with wild-type *oprD* | This study |
| Pae d1 Green oprD415* pUCP18-oprD | Pae d1 Green oprD415* complemented with pUCP18 plasmid carrying wild-type *oprD* | This study |
| Pae d1 Green oprD415* pUCP18-oprD415* | Pae d1 Green oprD415* complemented with pUCP18 plasmid carrying mutant *oprD* | This study |
| Pae d1 Green oprD415* pUCP18-empty | Pae d1 Green oprD415* complemented with empty pUCP18 plasmid | This study |
| pCasPA | Tc^r^, plasmid for bacterial expression of Cas9 nuclease and λ-Red recombination system | (1) |
| pACRISPR | Ap^r^, a sgRNA expression plasmid for targeting a specific sequence | (1) |
| pUCP18 | *Escherichia-Pseudomonas* shuttle vector | Laboratory storage |
| pET-30a | Expression plasmid for MexR-6×His fusion protein | Laboratory storage |

**Table S2. Primers used in this study.**

| **Primer** | **Sequence (5’-3’)** | **Description** |
| --- | --- | --- |
| **PCR primers** |  |  |
| 27-F | AGAGTTTGATCATGGCTCAG | For amplification of 16S rDNA for strain identification |
| 1492-R | CGGTTACCTTGTTACGACTT |  |
| mexRPE-F | GGAATTCCATATGAACTACCCCGTGAATCCCG | For amplification of *mexR* in protein expression |
| mexRPE-R | CCGCTCGAGAATATCCTCAAGCGGTTGCGCG |  |
| T7-F | TAATACGACTCACTATAGGG | Universal checking primers from the plasmid pET30a |
| T7-R | GCTAGTTATTGCTCAGCGG |  |
| mexREM-F | GGTAGTTCATTGGTTTGGCC | For amplification of the 291-bp *mexA*-*mexR* intergenic region for Electrophoretic Mobility Shift Assay (EMSA) |
| mexREM-R | GTTGCATAGCGTTGTCCTCA |  |
| Rpsl-F1 | GTCGACAAGAGCGACGTG | For amplification of the 142 -bp *rpsL* gene (negative control DNA) for EMSA |
| Rpsl-R1 | CGTACACGGCATACCTTACG |  |
| **RT-qPCR primers** | | |
| RT-MexB-F | CAGGTGTTCGGCTCGCA | For qualifying the expression of the efflux pump related gene *mexB* |
| RT-MexB-R | GATGATGGTGGCGTTGAGC |  |
| RT-MexA-F1 | TCCGAGGTTTCCGTCGAC | For qualifying the expression of the efflux pump related gene *mexA* |
| RT-MexA-R1 | CTTCCTGCAACTGCGCG |  |
| RT-Rpsl-F1 | GTCGACAAGAGCGACGTG | For qualifying the expression of the internal control gene *Rpsl* |
| RT-Rpsl-R1 | CGTACACGGCATACCTTACG |  |
| **Mutant construction primers** | | |
| mexRspacer-F | GTGGGCATCAGGTCGGGATTCACG | *mexR* spacers for gene deletion |
| mexRspacer-R | AAACCGTGAATCCCGACCTGATGC |  |
| mexRUP-F | TTTTGAGATCTGTCCATACCCATGGTCTAGAAACGACTTCGACGGCAGCTTCACC | For amplification of ~500 bp *mexR* upstream arm |
| mexRUP-R | ATTAGGTTTACTCGGCCAAACCACTTGAGGATATTTAAGAACATTCT |  |
| mexRDN-F | AGAATGTTCTTAAATATCCTCAAGTGGTTTGGCCGAGTAAACCTAAT | For amplification of ~500 bp *mexR* downstream arm |
| mexRDN-R | TCTGAATGGCGGGAGTATGAAAAGTCTCGAGTTGAGGATGATGCCGTTCACC |  |
| mexRKO-F | TCAAGATGGACCTCGGCCCG | For *mexR* knockout verification |
| mexRKO-R | TTAGCTCGATGGCCGGTTATCCA |  |
| SGRNAVF | TCTCGTTTGGATTGCAACTG | For pACRISPR plasmid verification |
| SGRNAVR | ACTTTCACCAGCGTTTCTGGG |  |
| Cas9-F | GCTCCCCGGTGAGAAGAAAA | For pCASPA plasmid verification |
| Cas9-R | TGAAGCTGATAGGGGAGCCT |  |
| mexRCP-F | CCGGAATTCATGAACTACCCCGTGAATCCCG | For amplification of *mexR* used in complement experiment |
| mexRCP-R | GCTCTAGATTAAATATCCTCAAGCGGTTGCGCG |  |
| LacZ-F | TCACACAGGAAACAGCTATGACC | For pUCP18 plasmid verification |
| LacZ-R | TTAAGTTGGGTAACGCCAGGG |  |
| oprD Spacer-F | GTGGAACTACTATTTCAACCGTGA | *oprD* spacers for gene replace |
| oprD Spacer-R | AAACTCACGGTTGAAATAGTAGTT |  |
| F-UPoprD | TTTTGAGATCTGTCCATACCCATGGTCTAGACAAACGCATTCGCCACAGACAACTCG | For amplification of ~500 bp Pae d1 *oprD* upstream arm |
| R-UPoprD | CCTAGAGCAAGACGTTTCCCGTTGAATATGGCTCATTGTGATTGCTCCTTTGGTTTTGAA |  |
| F-kanaoprD | TTCAAAACCAAAGGAGCAATCACAATGAGCCATATTCAACGGGAAACGTCTTGCTCTAGG | For amplification of *kanR* partial segment from pET30a |
| R-kanaoprD | GGTTTTTTCGTTGCCTGTCGGTCGACTGTAATGAAGGAGAAAACTCACCGAG |  |
| F-DNoprD | CTCGGTGAGTTTTCTCCTTCATTACAGTCGACCGACAGGCAACGAAAAAACC | For amplification of ~500 bp Pae d1 Green *oprD* downstream arm |
| R-DNoprD | TCTGAATGGCGGGAGTATGAAAAGTCTCGAGGGCCATCGATGATGAGGAGTCAG |  |
| F-UPoprD | TTTTGAGATCTGTCCATACCCATGGTCTAGACAAACGCATTCGCCACAGACAACTCG | For amplification of repair template containing *oprD*(W415*) or wild-type *oprD* flanked by 500-bp homologous arms |
| R-DNoprD | TCTGAATGGCGGGAGTATGAAAAGTCTCGAGGGCCATCGATGATGAGGAGTCAG |  |
| oprDkana-F | AAGAACTAGCCGTCACTGCG | For *oprD* replacement verification |
| oprDkana-R | GCGTTGCCGCCAAGAAGAAA |  |

**Table S3. Susceptibility of the resistant strains for the tested drugs after 7 days’ passage on drug-free agar plates.**

| Drugs | MIC (µg/mL) against: | | | |
| --- | --- | --- | --- | --- |
|  | Pae d14 Green | Pae d14 Brown | Pae d17 Green | Pae d17 Brown |
| Meropenem | 64 | 128 | 64 | 64 |
| Imipenem | 16 | 16 | 16 | 32 |


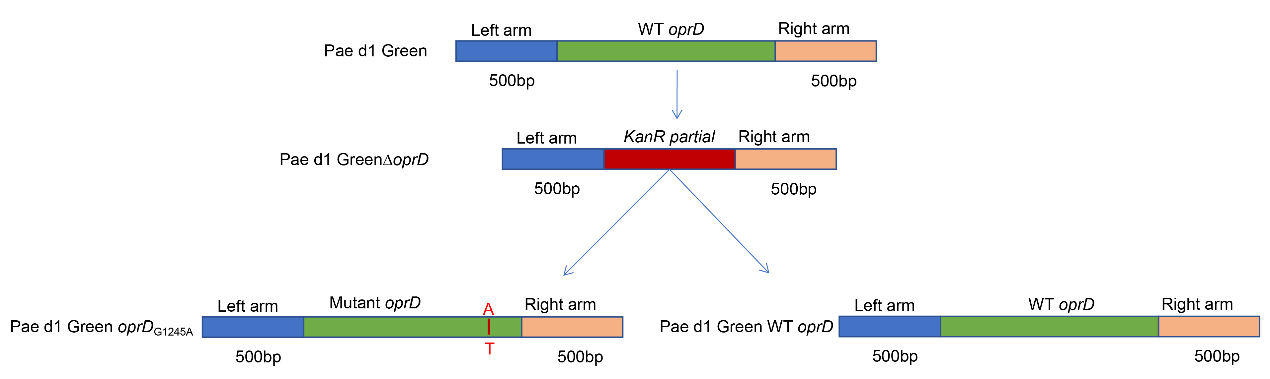


**FIG S1. Construction of Pae d1 Green *oprD* (G1245A) mutant.** Two-step gene replacement strategy was used, first replace the wild-type *oprD* gene with a kanR partial segment, and then replace the *kanR* segment with a wild-type or G1245A mutated *oprD* gene.


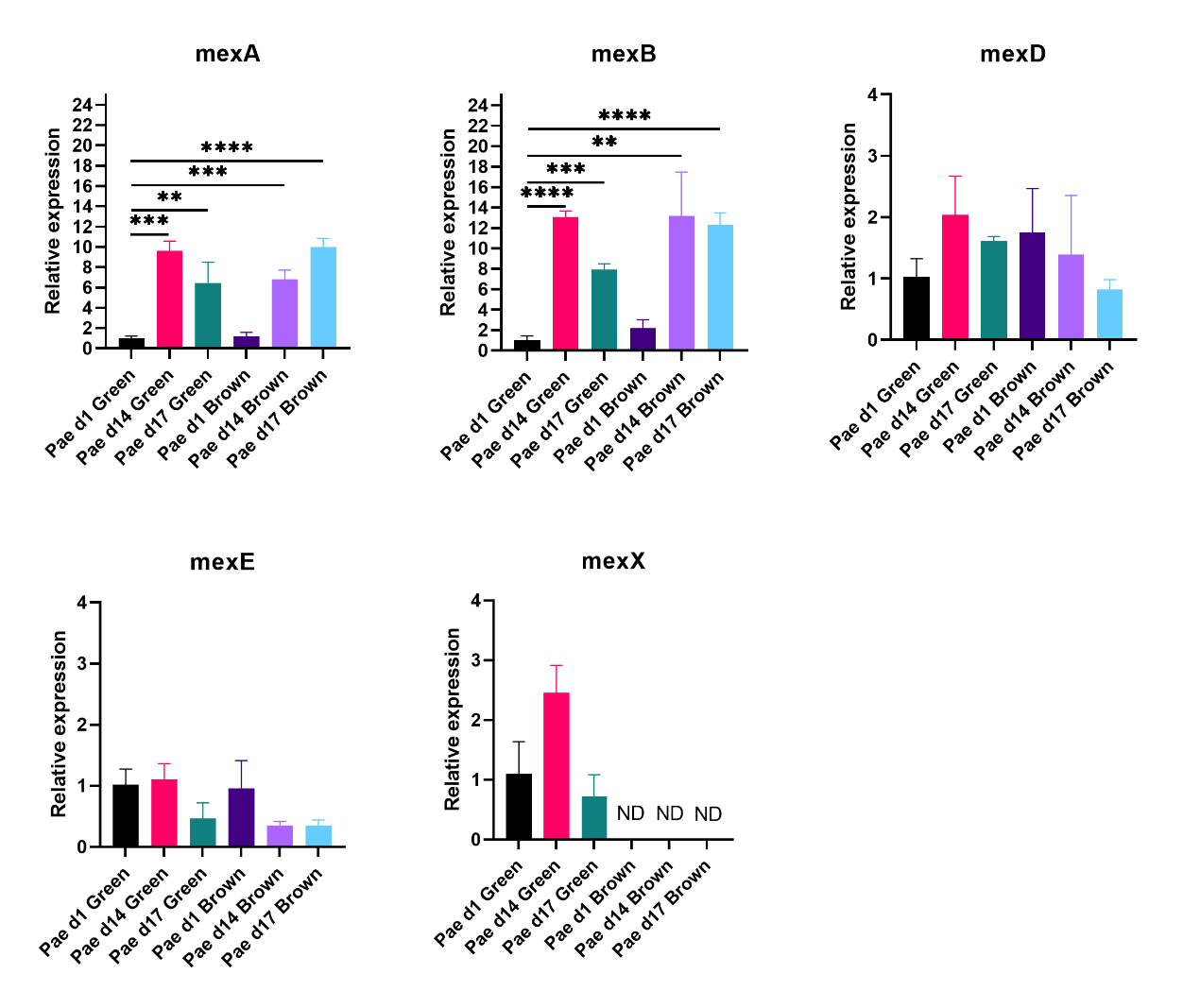


**FIG S2. The expressions of** **efflux pump related genes *mexA*, *mexB*, *mexD*, *mexE* and *mexX* of the strains compared with the Pae d1 Green.** The expression of *mexAB-oprM* efflux pump related genes *mexA* and *mexB* of resistant strains was significantly increased compared with the susceptible isolates. ND, undetectable due to deletion of *mexX* gene in all brown *P. aeruginosa*. **P*< 0.05, ***P*< 0.01, ****P*< 0.001, *****P*< 0.0001 using two-tailed *t-*test against mean fold change of susceptible isolates. At least 3 biological replicates were performed. Bar plots show mean fold change ± SD.

**References**

1. Chen W, Zhang Y, Zhang Y, Pi Y, Gu T, Song L, Wang Y, Ji Q. 2018. CRISPR/Cas9-based genome editing in *Pseudomonas aeruginosa* and cytidine deaminase-mediated base editing in *Pseudomonas* species. iScience 6:222–231.
